# Tick-Borne Encephalitis Vaccine Effectiveness and Impact in Slovenia, 2019–2024

**DOI:** 10.3390/pathogens15050503

**Published:** 2026-05-07

**Authors:** Jan Kordež, Frederick J. Angulo, Pingping Zhang, Alexander Davidson, Juanita Edwards, Lisa R. Harper, Andreas Pilz, Hannah Gould

**Affiliations:** 1 Medical and Scientific Affairs, Pfizer, SI-1000 Ljubljana, Slovenia; jan.kordez@gmail.com; 2 Medical Evidence Development, Bacterial Vaccines Real-World Evidence and Epidemiology, Pfizer, Collegeville, PA 19426, USA; 3 Medical Affairs Evidence Generation Statistics, Pfizer Research and Development, Collegeville, PA 19426, USA; pingping.zhang@pfizer.com; 4 Medical Evidence Development, Bacterial Vaccines Real-World Evidence and Epidemiology, Pfizer, New York, NY 19426, USA; alexander.davidson2@pfizer.com; 5 Medical Evidence Generation, Bacterial Vaccines Real-World Evidence and Epidemiology, Pfizer, Collegeville, PA 19426, USA; juanita.edwards@pfizer.com; 6 Medical Evidence Development, Vaccines, Integrated Evidence Generation, Pfizer, Collegeville, PA 19426, USA; lisa.r.harper@pfizer.com; 7 Global Medical Affairs, Bacterial and Vector-Borne Vaccines, Pfizer Biopharma, 1210 Vienna, Austria; andreas.pilz@pfizer.com; 8 Global Medical Affairs, Bacterial and Vector-Borne Vaccines, Pfizer Research and Development, New York, NY 10001, USA; hannah.gould@pfizer.com

**Keywords:** vector-borne disease, epidemiology, surveillance, prevention, public health, vaccine

## Abstract

Slovenia has a high tick-borne encephalitis (TBE) incidence and TBE vaccination is recommended for all residents ≥1 year-of-age. Despite widespread TBE vaccine use in Slovenia, TBE vaccine effectiveness (VE) has not been reported for Slovenia. We defined individuals who received ≥3 TBE vaccine doses in accordance with the recommended vaccination schedule as fully vaccinated. Using the screening method, TBE VE was determined by comparing the proportions of TBE cases and survey respondents who were fully vaccinated. TBE incidence and VE were used to estimate the number of TBE cases prevented by TBE vaccination. Of 637 surveillance-reported TBE cases in 2019–2024, 332 (52.1%) had a known vaccination history. Of these 332 TBE cases, 322 (97.0%) were unvaccinated and 10 (3.0%) received ≥1 doses of a TBE vaccine. Among 25,242 persons surveyed with known TBE vaccination history, 57.9% were unvaccinated. VE of ≥3 TBE doses for the prevention of TBE was 93.4% (84.0–97.3%). TBE vaccination prevented 426 TBE cases in 2019–2024. If the entire population was vaccinated against TBE, vaccination would have prevented 994 TBE cases in 2019−2024. TBE vaccination in Slovenia was highly effective, preventing hundreds of TBE cases a year. Most of the Slovenian population, however, is not vaccinated against TBE. To prevent additional TBE cases in Slovenia, further efforts are needed to enhance TBE vaccine uptake.

## 1. Introduction

Tick-borne encephalitis (TBE) is an infection by the tick-borne encephalitis virus (TBEV), resulting in signs and symptoms of central nervous system inflammation (e.g., meningitis, encephalitis, encephalomyelitis) [1,2]. TBEV is transmitted to humans most commonly by the bite of an *Ixodes* tick and rarely by consumption of unpasteurized dairy products [1,2]. TBE can be life-threatening and can result in long-term neurological sequalae [2,3]. TBE is endemic in several European countries including Slovenia, where the public health threat of TBE is growing, as evidenced by an increasing incidence and expansion of TBE-endemic areas [2,3,4,5,6].

In 2022, thirty European countries reported 3516 TBE cases to the European Centre for Disease Prevention and Control (ECDC), resulting in an incidence of 0.8/100,000 population per year (PPY) [7]. Of the TBE cases reported to ECDC, 158 (4.5%) were from Slovenia. The TBE incidence in Slovenia was 2.9/100,000 PPY in 2022, which was the fourth highest TBE incidence among ECDC-reporting countries. The clinical severity of TBE is indicated by the observation that most patients with a surveillance-reported TBE case in Slovenia are hospitalized [5].

Vaccination provides the most effective protection against TBE [8]. TBE vaccination in Slovenia has been recommended for individuals at high risk of occupational exposure to *Ixodes* ticks since 1986 and for all residents of, as well as travelers to, TBE-endemic areas in Slovenia since 1991 [3,6,9]. TBE vaccination has also been included in the national vaccination program of Slovenia, with vaccination paid for by compulsory health insurance, since 2019 [3]. Furthermore, as of March 2025, health insurance is required to cover TBE vaccination for children born after 2016 and adults born in 1970–1980 who have not yet received three TBE vaccine doses [10]. Two TBE vaccines (FSME-IMMUN^®^ [Pfizer Europe, Brussels, Belgium] and Encepur^®^ [Bavarian Nordic, Hellerup, Denmark]) have been approved for use in countries in Europe since 1976 and 1991, respectively [1]. FSME-IMMUN^®^ was the most widely used TBE vaccine in Slovenia in 2019–2024 [5]. The recommended vaccination schedule for TBE vaccines in Slovenia is a three-dose primary series (at months 0, 1, and 9–12) followed by the first booster 3 years after the primary series, with subsequent boosters every 3 years for persons ≥ 60 years of age and every 5 years for persons < 60 years of age [9].

A 2020 survey of the general population in Slovenia reported that 39% of the Slovenian population had received at least one dose of a TBE vaccine [11]. However, additional surveys indicated that the actual TBE vaccine coverage of the general population in Slovenia may be <20% [12,13]. For comparison, a 2022 survey of the general population in Austria, with whom Slovenia shares a border and has comparable TBEV endemicity, reported a TBE vaccine uptake by the general population of 81% [11]. Factors contributing to the lower TBE vaccine uptake in Slovenia compared with Austria include limited awareness of the risk for acquiring TBEV infection or the severity of TBE and vaccine costs [14].

Following the widespread introduction of a TBE vaccine in Austria in 1979, the incidence of TBE cases in Austria decreased markedly, with case numbers declining rapidly [15]. Previous studies have reported estimates of TBE vaccine effectiveness (VE) of ≥95% against TBE in several European countries [16]. However, estimates of TBE VE and of the vaccination impact (i.e., the number of TBE cases prevented by TBE vaccination) are not available for Slovenia. Therefore, the aim of this study was to provide the first estimates of TBE VE and assess the impact of TBE vaccination in Slovenia.

## 2. Materials and Methods

### 2.1. Study Design and Population

Slovenia is a central European country which is divided into twelve statistical regions. The study population included all individuals in Slovenia ≥ 1 year of age. Slovenian census data are available from the Statistical Office of the Republic of Slovenia (SURS) (https://www.stat.si/statweb/en, accessed on 4 December 2025). According to SURS, the average population ≥ 1 year of age in Slovenia in 2019–2024 was 2,087,122. The study analyzed nationwide surveillance data on TBE cases that are routinely collected by the National Institute of Public Health (NIJZ) (https://nijz.si/en/nijz/, accessed on 4 December 2025). For our study, the available information on surveillance-reported TBE cases, provided upon request from NIJZ, included TBE vaccination history, but this did not include hospitalization and other patient outcome information. Only aggregate data with no personal identifiers were utilized; therefore, no ethical review committee approval was required when obtaining the data for the analysis.

### 2.2. Case Definition and Case Data

TBE has been a notifiable disease in Slovenia since 1977 [17]. Clinical laboratories and physicians report laboratory-diagnosed TBE cases to NIJZ. In accordance with ECDC case definitions [18], a laboratory-confirmed TBEV infection is a patient with anti-TBEV IgG and IgM antibodies in blood, seroconversion or a four-fold increase in anti-TBEV antibodies in paired serum samples, or anti-TBEV IgM antibodies in CSF.

### 2.3. Vaccine Uptake

Online household surveys, which followed standard survey procedures used by commercial marketing companies to derive national prevalence estimates, were conducted each year by Ipsos GmbH (Hamburg, Germany) during 2019–2024 to derive nationwide estimates of TBE vaccination knowledge, attitudes, and practices of individuals selected from the general population [11,19]. Participants were recruited to participate in the online general population surveys based on publicly available Eurostat data (https://ec.europa.eu/eurostat, accessed on 4 December 2025) using sampling proportions for gender, region, occupational status, and household size. Survey respondents (one per household) were 18–65 years of age and provided information for all household members ≥ 1 year of age. Surveys took 5–15 min and gathered information on age, gender, geographic area of residence, and TBE vaccination history. Respondents consulted vaccination cards for TBE vaccination history. Survey results were weighted using national census data to derive national prevalence estimates.

### 2.4. Statistical Analysis

A person was considered fully vaccinated if he or she received ≥3 TBE vaccine doses and was not late in receiving the next dose in accordance with the vaccine schedule. A person was considered partially vaccinated if he or she received one or two TBE vaccine doses, or received ≥3 doses but was late in receiving the next dose. VE against TBE was estimated under two scenarios. In a base-case scenario, TBE cases who received ≥3 doses but did not report the vaccination dates were classified as fully vaccinated. In a sensitivity analysis, these cases were classified as partially vaccinated. VE against TBE for the period 2019–2024 was estimated using the screening method [20]. This method compared the proportion of TBE cases who were fully vaccinated (PCV) with the proportion of persons in the general population surveys who were fully vaccinated (PPV), and excluded persons who were partially vaccinated. VE was calculated using the following formula: VE = 1 − [PCV/(1 − PCV)]/[PPV/(1 − PPV)]. Estimates of VE were calculated with 95% confidence intervals (CIs).

To estimate the number of TBE cases prevented by vaccination, the number of surveillance-reported TBE cases from 2019 to 2024 was compared with the estimated number of cases that would have occurred if no one in the population had been vaccinated. The expected number of cases in the absence of vaccination was estimated by multiplying the Slovenian population by the incidence of TBE in the unvaccinated population. This incidence was derived from the number of surveillance-reported TBE cases who were unvaccinated (estimated by extrapolating vaccination history among cases with known status) and the size of the unvaccinated population (estimated from surveillance data and population surveys). The difference between the expected number of cases without vaccination and the observed number of cases from 2019 to 2024 was used to estimate the number of TBE cases prevented by vaccination. Finally, the estimated number of cases that would have occurred without vaccination was multiplied by the base-case VE estimate to estimate the number of cases that would have been prevented if the entire population had been vaccinated.

## 3. Results

### 3.1. Surveillance-Reported TBE Cases

**Figure 1:**
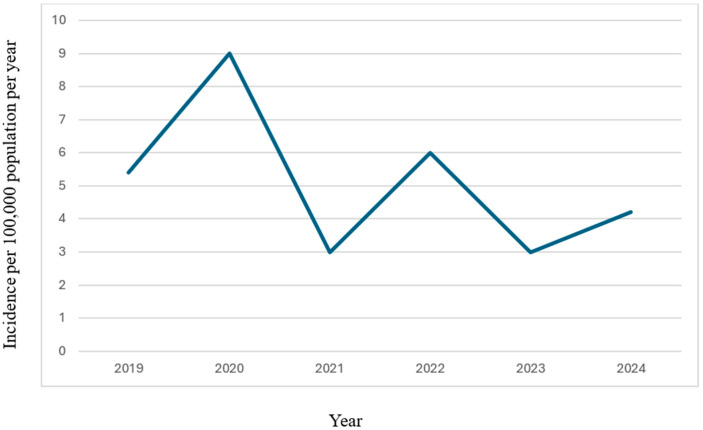
Incidence (per 100,000 population per year) of surveillance-reported tick-borne encephalitis cases in Slovenia, 2019–2024.

**Figure 2:**
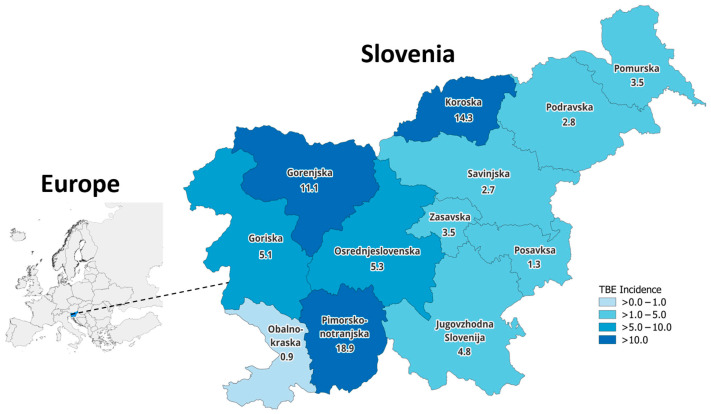
Map of Europe with the incidence of surveillance-reported tick-borne encephalitis cases (per 100,000 population per year) by region in Slovenia, 2019–2024.

There were 637 surveillance-reported TBE cases in Slovenia in 2019–2024, including 89 surveillance-reported TBE cases in 2024. From 2019 to 2024, the incidence of TBE was 5.1/100,000 PPY, with a peak in 2020 (9.0/100,000 PPY) (Figure 1). Of the 637 TBE cases, 62 (9.7%) TBE cases were in the 1–15 years-of-age group, 357 (56.0%) in the 16–59 years-of-age group, and 218 (34.2%) in the ≥60 years-of-age group. TBE incidence was 3.1/100,000 PPY in children 1–15 years of age, 5.1/100,000 PPY in persons 16–59 years of age, and 6.2/100,000 PPY in persons ≥ 60 years of age. Incidence also varied by geographic area, with the highest incidence in Primorsko-notranjska (18.9/100,000 PPY) and Koroška (14.3/100,000 PPY), and the lowest incidence in Obalno–Kraška (0.9/100,000 PPY) (Figure 2).

TBE vaccination history was available for 332 (52.1%) of the 637 surveillance-reported TBE cases (Table 1). Of these 332 TBE cases, 322 (97.0%) were unvaccinated and 10 (3.0%) received ≥1 doses of a TBE vaccine, including three (30.0%) with unknown dosing information (partially vaccinated). Of the seven TBE cases who received a TBE vaccine with a known dosing information, one (14.3%) received 2 doses (partially vaccinated) and six (85.7%) received ≥3 doses. Of the six TBE cases who received ≥3 doses, three (5%) reported the dates of vaccination; of whom, one (33.3%) was late for the next dose (partially vaccinated), and two (66.7%) received doses in accordance with the vaccination schedule (fully vaccinated). The three TBE cases who received ≥3 doses but did not report the dates of vaccination were assumed to be fully vaccinated in the base-case scenario and assumed to be partially vaccinated in the sensitivity analysis. Therefore, of the 332 TBE cases with vaccination history, five (1.5%) cases were fully vaccinated with ≥3 doses in the base-case scenario and two (0.6%) were fully vaccinated with ≥3 doses in the sensitivity analysis.

### 3.2. General Population Survey

From 2019 to 2024, 26,075 individuals participated in the general population surveys (4736 in 2019, 5188 in 2020, 4716 in 2021, 4762 in 2022, 4773 in 2023, and 4900 in 2024). Of the survey participants, 25,242 (86.8%) provided information on TBE vaccination history (Table 1). Among the people providing information on TBE vaccination history, 14,619 (57.9%) were unvaccinated and 10,623 (42.1%) had received ≥1 doses of a TBE vaccine. In the twelve regions of Slovenia, among the people providing information on TBE vaccination history, the proportion of the population that was unvaccinated ranged from 67.0% in Zasavska and 64.9% in Obalno–Kraška to 49.3% in Pirmorsko-notranjska and 46.5% in Koroška. Nationwide, of the 10,623 who received a TBE vaccine, 4780 (45.0%) had a known number of doses and dates of vaccination, and 5843 (55.0%) had an unknown number of doses or dates of vaccination. Among the 4780 with a known number of doses and dates of vaccination, 3235 (67.7%) were partially vaccinated and 1545 (32.3%) were fully vaccinated. Therefore, the estimates of TBE vaccine uptake by the general population in Slovenia from 2019 to 2024 was 57.9% unvaccinated, 28.5% partially vaccinated, and 13.6% fully vaccinated.

Using the results from the general population, the estimated number of unvaccinated persons ≥1 year of age in Slovenia was 1,208,235 per year in 2019–2024; using the extrapolated data from the surveillance-reported TBE cases, the estimated incidence of TBE in the unvaccinated population was 8.5/100,000 PPY in 2019–2024 (compared to 5.1/100,000 PPY in the overall population).

### 3.3. TBE Vaccine Effectiveness

Under the base-case scenario, the estimated VE of ≥3 TBE vaccine doses against TBE in 2019–2024 was 93.4% (95% CI 84.0–97.3) (Table 2). Under the sensitivity analysis scenario, the estimated VE of ≥3 TBE vaccine doses against TBE in 2019–2024 was 97.4% (95% CI 89.4, 99.3).

### 3.4. TBE Cases Prevented by Vaccination

In the absence of TBE vaccination, we estimated that there would have been 1064 TBE cases in Slovenia from 2019 to 2024. When compared with the 637 observed TBE cases, this indicates that TBE vaccination prevented 426 TBE cases in Slovenia over this period. With an observed VE of ≥3 vaccine doses of 94.3% (95% CI 84.0–97.3) for the prevention of TBE cases, receipt of ≥3 doses of a TBE vaccine by the entire population of Slovenia would have prevented 994 TBE cases in 2019–2024.

## 4. Discussion

TBE vaccines were highly effective in the prevention of TBE and had a substantial impact on the burden of TBE in Slovenia in 2019–2024. During the 6 year study period, receiving ≥3 doses of a TBE vaccine in accordance with the recommended vaccination schedule prevented approximately over 400 TBE cases. The high effectiveness of TBE vaccines and the public health impact of TBE vaccination observed in our study in Slovenia are consistent with other observational studies conducted in Austria, the Czech Republic, Estonia, Germany, Latvia, Lithuania, Sweden, and Switzerland [16,21,22]. Furthermore, the high effectiveness of FSME-IMMUN^®^ has been demonstrated in studies in Austria, Germany, and Latvia [16].

Despite the effectiveness of TBE vaccines, there were 637 surveillance-reported TBE cases in Slovenia during the study period. TBE is endemic in almost the entire country of Slovenia [5], and Slovenia has among the highest reported TBE incidence in Europe [7]. *Ixodes* ticks that transmit TBEV are frequently found in forests and >60% of Slovenia is forested; a correlation between the forested areas and TBE incidence in Slovenia has been demonstrated [23]. Importantly, in our study, we demonstrate that almost all surveillance-reported TBE cases in Slovenia occurred among individuals who were unvaccinated. Furthermore, other published research has estimated that the TBE incidence among unvaccinated individuals in Slovenia was 7.7 per 100,000 population per year [24]. Despite the high TBE incidence in Slovenia and the propagation of TBE vaccination recommendations for Slovenian residents, we found that only 43.1% of the general population had received at least 1 dose of a TBE vaccine. Although this estimate of TBE vaccine uptake by the general population is higher than in previous surveys [11,19], these results highlight that TBE vaccine uptake in Slovenia is suboptimal and suggests that low uptake is an important contributing factor to the high TBE incidence in the country. Further efforts are needed to increase TBE vaccine uptake in Slovenia to prevent TBE, a potentially disabling and life-threatening disease [3,4,5,14,17].

A limitation of this study is that the TBE vaccination history could not be determined for 13.2% of general population survey participants and 47.9% of surveillance-reported TBE cases; we do not know if individuals with missing vaccination histories are more or less likely to have received a TBE vaccine, and if this differs between survey participants and TBE cases. Another limitation is that the VE estimates were derived via the screening method which compared the vaccination history of patients with TBE with that of survey responders selected from the general population. Potential selection bias is a concern as individuals who were TBE vaccinated may have been more likely to participate in the population survey than individuals who were unvaccinated. Potential misclassification bias is also a concern since different approaches were used to determine the vaccination history of TBE cases and of the surveyed general population. Although the direction of the selection and misclassification bias in the screening method approach is unknown, two recent studies estimated TBE VE using both a matched case–control analysis and the screening method and found lower, and thus more conservative, TBE VE estimates using the screening method, which suggests that the bias using the screening method may be toward the null resulting in lower VE estimates [25,26]. Our study has other limitations, including inherent uncertainties, in our approach for estimating the number of TBE cases prevented by TBE vaccination and estimating the number of TBE cases that would be prevented if everyone was TBE vaccinated. In addition, our study was unable to estimate brand-specific VE because the surveillance-reported TBE cases in our study may have received either of the two TBE vaccines available in Europe.

## 5. Conclusions

This first published study of TBE VE in Slovenia showed that TBE vaccination was highly effective for the prevention of TBE cases. Vaccination prevented hundreds of TBE cases despite the relatively low TBE vaccine uptake and compliance by the general population in Slovenia. To prevent additional TBE cases in Slovenia, enhanced efforts to increase TBE vaccine uptake are needed.

## Figures and Tables

**Table 1:** Tick-borne encephalitis (TBE) vaccination history of surveillance-reported TBE cases and general population survey respondents in Slovenia, 2019–2024.

	Surveillance-Reported TBE Cases *		General Population Survey Respondents
	Base Case	Sensitivity Analysis	
*Among TBE cases/survey respondents*	n = 637	n = 637	n = 29,075
Known vaccination history	332 (52.1%)	332 (52.1%)	25,242 (86.8%)
Unknown vaccination history	305 (47.9%)	305 (47.9%)	3833 (13.2%)
*Among those with known* *vaccination history*	n = 332	n = 332	n = 25,242
Vaccinated with ≥1 dose	10 (3.0%)	10 (3.0%)	10.623 (42.1%)
Unvaccinated	322 (97.0%)	322 (97.0%)	14,619 (57.9%)
*Among those vaccinated* *with ≥1 dose*	n = 10	n = 10	n = 10,623
Known vaccine doses	7 (70.0%)	7 (70.0%)	4780 (45.0%)
Unknown vaccine doses	3 (30.0%)	3 (30.0%)	5843 (55.0%)
*Among those vaccinated with ≥1 dose with known vaccine doses*	n − 7	n = 7	n = 5843
Partially vaccinated	2 (28.6%)	5 (71.4%)	3235 (67.7%)
Fully vaccinated	5 (71.4%)	2 (28.6%)	1545 (32.3%)
*Estimates of vaccine uptake*			
Fully vaccinated	97.0%	97.0%	13.6%
Partially vaccinated	1.5%	2.4%	28.5%
Unvaccinated	1.5%	0.6%	57.9%

* In the base case, three surveillance-reported TBE cases who received ≥3 TBE vaccine doses with unknown date of vaccination were assumed to be vaccinated in accordance with the vaccination schedule (fully vaccinated). In the sensitivity analysis, these three TBE cases were assumed not be vaccinated in accordance with the vaccination schedule (partially vaccinated).

**Table 2:** Vaccine effectiveness (VE) of three or more doses of a tick-borne encephalitis (TBE) vaccine against TBE under the base-case scenario and sensitivity-analysis scenario in Slovenia, 2019–2024.

Number of Surveillance -Reported TBE Cases	Number (Percent) of Surveillance- Reported TBE Cases with Known Vaccination History	Number (Percent) of Surveillance- Reported TBE Cases with Known Vaccination History Who Were Unvaccinated	Sensitivity-Analysis Scenario		Base-Case Scenario	
			Number of Fully Vaccinated Cases	VE (95% CI)	Number of Fully Vaccinated Cases	VE (95% CI)
637	332 (52.1)	322 (97.0)	2	97.4 (89.4, 99.3)	5	93.4 (84.0–97.3)

## Data Availability

The data supporting this study’s findings are available from the corresponding author upon reasonable request.

## Abbreviations

The following abbreviations are used in this manuscript:

ECDC	European Centre for Disease Prevention and Control
NIJZ	National Institute of Public Health
PCV	Proportion of tick-borne encephalitis cases who were fully vaccinated
PPY	Per population per year
PPV	Proportion of persons in the general population surveys who were fully vaccinated
SURS	Statistical Office of the Republic of Slovenia
TBE	Tick-borne encephalitis
TBEV	Tick-borne encephalitis virus
VE	Vaccine effectiveness

## References

[1] Lindquist L., Vapalahti O. (2008). Tick-borne encephalitis. Lancet.

[2] Süss J. (2011). Tick-borne encephalitis 2010: Epidemiology, risk areas, and virus strains in Europe and Asia-an overview. Ticks Tick Borne Dis..

[3] Šmit R. (2019). Reviewing estimates of the burden in disability-adjusted life years (DALYs) of tick-borne encephalitis in Slovenia. Expert Rev. Pharmacoecon. Outcomes Res..

[4] Fafangel M., Cassini A., Colzani E., Klavs I., Grgič Vitek M., Učakar V., Muehlen M., Vudrag M., Kraigher A. (2017). Estimating the annual burden of tick-borne encephalitis to inform vaccination policy, Slovenia, 2009 to 2013. Euro Surveill..

[5] Simonović Z., Učakar V., Praprotnik M., Dobler G., Erber W., Bröker M., Chitimia-Dobler L., Schmit H.J. (2025). TBE in Slovenia. TBE Book.

[6] Angulo F.J., Halsby K., Davidson A., Ravikumar S., Pilz A., Stark J.H., Moïsi J.C. (2024). Publicly available surveillance data on tick-borne encephalitis in Europe, 2023. Ticks Tick Borne Dis..

[7] European Centre for Disease Prevention and Control (ECDC) (2024). Tick-Borne Encephalitis Annual Epidemiological Report for 2022.

[8] WHO Publication (2011). Vaccines against tick-borne encephalitis: WHO position paper—Recommendations. Vaccine.

[9] Grgič-Vitek M., Avšič-Županc T., Klavs I. (2010). Tick-borne encephalitis after vaccination: Vaccine failure or misdiagnosis. Vaccine.

[10] Slovenian National Institute of Public Health (NIJZ) Recommendations for Vaccination Against Tick-Borne Meningoencephalitis. https://nijz.si/wp-content/uploads/2014/05/TBE-Vaccination-information-leaflet.pdf.

[11] Pilz A., Erber W., Schmitt H.J. (2023). Vaccine uptake in 20 countries in Europe 2020: Focus on tick-borne encephalitis (TBE). Ticks Tick Borne Dis..

[12] Grgič-Vitek M. (2019). Self-reported vaccination coverage against tick-borne encephalitis in Slovenia. Ticks Tick Borne Dis..

[13] NIJZ (2018). Neenakosti v Zdravju v Sloveniji v Času Ekonomske Krize (Health Inequalities in Slovenia in Time of Economic Crisis).

[14] Grgic-Vitek M., Klavs I. (2012). Low coverage and predictors of vaccination uptake against tick-borne encephalitis in Slovenia. Eur. J. Public Health.

[15] Kunz C. (2003). TBE vaccination and the Austrian experience. Vaccine.

[16] Angulo F.J., Zhang P., Halsby K., Kelly P., Pilz A., Madhava H., Moïsi J.C., Jodar L. (2023). A systematic literature review of the effectiveness of tick-borne encephalitis vaccines in Europe. Vaccine.

[17] Grgič-Vitek M., Klavs I. (2011). High burden of tick tick-borne encephalitis in Slovenia—Challenge for vaccination policy. Vaccine.

[18] (2012). European Commision. Commission implementing decision of 8 August 2012 amending Decision 2002/253/EC laying down case definitions for reporting communicable diseases to the Community network under Decision No 2119/98/EC of the European Parliament and of the Council. Off. J. Eur. Union.

[19] Erber W., Schmitt H.J. (2018). Self-reported tick-borne encephalitis (TBE) vaccination coverage in Europe: Results from a cross-sectional study. Ticks Tick Borne Dis..

[20] Farrington C.P. (2023). Estimation of vaccine effectiveness using the screening method. Int. J. Epidemiol..

[21] Palmborg A., Angulo F.J., Zhang P., Pilz A., Stark J., Moïsi J.C., Jodar L. (2025). Tick-borne encephalitis vaccine uptake, effectiveness, and impact in Sweden from 2018 to 2022. Sci. Rep..

[22] Angulo F.J., Zhang P., Žygutienė M., Zavadska D., Aimla K., Kivistik A., Griskevica A., Vadapaliene A., Bormane A., Harper L.R. (2025). Tick-borne encephalitis vaccine effectiveness and public health impact in the Baltic countries of Estonia, Latvia, and Lithuania, 2019–2023. IJID Reg..

[23] Knap N., Avšič-Županc T. (2015). Factors affecting the ecology of tick-borne encephalitis in Slovenia. Epidemiol. Infect..

[24] Halsby K., Davidson A., Davis J., Zens K., Dobler G., Pilz A., Angulo F.J., Zhang P., Kelly P.H., Stark J.H. (2025). Incidence of tick-borne encephalitis in unvaccinated populations across Europe (2020–2023). Int. J. Infect. Dis..

[25] Nygren T.M., Pilic A., Böhmer M.M., Wagner-Wiening C., Wichmann O., Harder T., Hellenbrand W. (2022). Tick-borne encephalitis vaccine effectiveness and barriers to vaccination in Germany. Sci. Rep..

[26] Zens K.D., Altpeter E., Wymann M.N., Mack A., Baer N.B., Haile S.R., Steffen R., Fehr J.S., Lang P. (2024). A combined cross-sectional analysis and case-control study evaluating tick-borne encephalitis vaccination coverage, disease and vaccine effectiveness in children and adolescents, Switzerland, 2005 to 2022. Euro Surveill..

